# Buffalo Academy of Medicine

**Published:** 1903-03

**Authors:** N. L. Burnham


### Buffalo Academy of Medicine.

*Section on Medicine, January 13, 1903.*

Reported by N. L. BURNHAM, M. D., Secretary.

The regular meeting of the section on medicine, of the Buffalo Academy of Medicine, was held at the Academy Rooms, Public Library Building, on Tuesday evening, January 13, 1903, the chairman, Dr. A. E. Woehnert, presiding.

The minutes of the previous meeting were read and approved.

The first paper of the evening was read by Dr. A. J. Colton, entitled,

### INFANTILE ECLAMPSIA.

### DISCUSSION.

Dr. I. M. Snow had never seen convulsions caused by either phimosis or dentition. He considers gastrointestinal disorders the most common cause. He reported a case of a seven months' old child whom he was called to see, which had a rectal temperature of 109.4° and convulsions. The child was treated with ice, viz.: ice pack, ice cold rectal irrigation, the high temperature being considered the cause of the convulsions. The convulsions ceased with the decline of the temperature. The case proved to be one of syphilitc meningitis.

Dr. C. S. Jones regards intestinal toxemia as the most common cause of infantile convulsions. He reported a case of capillary bronchitis with convulsions in a child six months old. He thinks the toxemia, resulting from the swallowed sputum, the factor in producing convulsions in this case. He considers hot applications in the form of hot moist cloths to feet and legs with cold to the head better than the hot bath in many cases. The child remains in bed and rests.

Dr. F. Thoma reported a case of a child three years of age in perfect health being suddenly seized with convulsions, and although treatment was begun at once, directed particularly toward the gastrointestinal canal, the child died in two hours. He could not make a diagnosis.

Dr. Schroeter had seen cases similar to that of Dr. Thoma's and considers them as due to meningitis. They are difficult to diagnosticate.

Dr. A. L. Benedict regarded intestinal irrigation as an efficient method of treating convulsive seizures in infants, but questions whether the irrigation relieves as a result altogether of toxins removed. We cannot relieve the whole intestinal canal by a single irrigation. May it not be that the convulsive seizure is due to an effort at expulsion on the part of the intestine and the assistance we render by irrigation causes convulsions to cease?

Dr. Colton, in closing the discussion, remarked that he considered the suddenness with which high temperature developed to be a great factor in producing convulsions.

The second paper of the evening was read by Dr. Irving M. Snow, entitled Injuries and infections of newly-born children, to be published at a later day.

---

*Stated Meeting, January 20, 1903.*

Reported by F. W. McGUIRE, M. D., Secretary.

The meeting was called to order by the president, Dr. Herman E. Hayd, at 9 p. m. The minutes of the last stated meeting, held November 3, 1902, were read and approved.

The council recommended the election of Drs. George F. Mills, Edward G. Aldrich and George Armstrong, all of the Buffalo State Hospital. They were duly balloted for and elected members of the Academy.

A communication from the officers of the New York State Nurses' Association, with reference to a bill before the present legislature, regarding the registration of graduate nurses, was read and on motion it was laid over to be considered at a special meeting.

The president appointed Drs. Grover W. Wende, John A. Pettit and William Meisburger, a committee to prepare resolutions regarding the death of Dr. Jacob M. Kraus.

The program of the evening was provided by the pathological section. It consisted of a paper read by Dr. MATTHEW D. MANN, entitled

SURGICAL TREATMENT OF PUERPERAL INFECTION.

The paper was discussed by Drs. Frederick, Van Peyma, Schroeter, O'Gorman and Hayd.

Dr. POHLMAN made a few remarks asking for a report of the committee on St. Mary's Maternity Hospital and Orphan Asylum.

Dr. MARSHALL CLINTON introduced the following resolution:

*Resolved*, That the Academy will not consider the charges, as it is a society for scientific discussion and not a medical police court.

It was moved as an amendment that the committee be discharged by the Academy. Carried.

Attendance, 48. Adjourned at 10.30 p. m.

